# Editorial: Child Sexual Abuse: The Interaction Between Brain, Body, and Mind

**DOI:** 10.3389/fpsyg.2019.02558

**Published:** 2019-11-29

**Authors:** Rachel Lev-Wiesel, Denise Saint Arnault

**Affiliations:** ^1^The Emili Sagol Creative Arts Therapies Research Center, University of Haifa, Haifa, Israel; ^2^Faculty of Social Welfare and Health Sciences, Emili Sagol Creative Arts Therapies Research Center, University of Haifa, Haifa, Israel; ^3^Department of Health Behavior and Biological Sciences, School of Nursing, University of Michigan, Chicago, IL, United States

**Keywords:** childhood sexual abuse (CSA), mind - body, treatmeat outcome, outcome, brain

Child sexual abuse (CSA) is a prevalent phenomenon worldwide (e.g., Stoltenborgh et al., 2011). Evidence shows that there is a strong relationship between child sexual abuse and long-term adverse mental and physical health consequences (e.g., Hall and Hall, 2011). However, less than 10% of children disclose or report the sexual abuse (Alaggia et al., 2019). Moreover, many victimized children do not receive help and are exposed to further victimization (Gagnier and Collin-Vézina, 2016). Sadly, research findings have also shown that the more severe the abuse, the higher the reluctance to disclose (e.g., Lev-Wiesel and First, 2018). Of investigations opened into possible child sexual victimization, about 85% ended in closing the case, either because of a lack of collaborating evidence (forensic evidence or witnesses) or due to the alleged victimized child's inability to provide a coherent testimony as required by the judicial system (e.g., Easton, 2013).

Despite the vast array of studies focusing on the different aspects of CSA, the current state of the art indicates a dearth of identification and detection tools, investigation protocols, and treatment model interventions. For example, although evidence indicating that the etiologic abnormalities of psychological trauma caused by CSA and traumatic brain injury (TBI) involve similar neurobiological systems and produce overlapping clinical syndromes (Sherin, 2011), the treatment modalities that are used to help CSA trauma survivors are merely psychological, sometimes accompanied with pharmacological treatment. From a trauma recovery perspective, considering the wide spectrum of physical, emotional, cognitive, social, and spiritual sequela after CSA, one wonders if treatment that is mainly psychological can be sufficient. The fact that millions of people with a history of CSA suffer from the deleterious effects of these experiences throughout their lives, even if given psychotherapy, raises questions about the extent to which it is possible to recover from CSA trauma and whether therapeutic goals should be limited to symptom reduction or posttraumatic growth. Moreover, concerning the criminal and judicial procedures in cases of CSA, looking to additional forensic evidence such as advanced brain scanning as collaborative evidence in cases of CSA might be crucial in helping victims, especially minors. From a psychosocial perspective, considering that abuse is often intergenerational, we must wonder about the impact of self-disclosure and treatment from an intergenerational perspective. From a theoretical perspective, many questions remained unanswered. For example, should CSA be considered a physiological trauma due to its impact on brain functionality? Do different traumas affect different brain areas?

This specific issue aims to broaden our knowledge about the impact of childhood sexual abuse trauma, its intergenerational aspects, and innovative treatment approaches by examining some of these questions. This issue consists of five papers. The first paper, authored by Saint Arnault and Sinko, compares identity, distress, and positive health outcomes of CSA survivors with women who have experienced unwanted sexual experiences (USE). Their findings indicate that whereas survivors of both CSA and USE showed Post-Traumatic growth scores, survivors of unwanted sexual experiences alone had significantly higher means for Sense of Coherence and Self-Compassion scores. This finding seems to support the evidence that trauma recovery experiences are different depending on the types of trauma and that trauma recovery is more complex and multifaceted than simply being a matter of psychological health (in this case, including identity, physical, and physical dimensions). Two papers examine the intergenerational aspects of CSA. Borelli et al. studied the associations between mothers' and children's histories of childhood sexual abuse (CSA) and children's psychiatric outcomes using an intergenerational perspective. They examined whether maternal reflective functioning about their trauma was associated with a lower likelihood of children being exposed to abuse (among children of CSA-exposed others). Their key finding is that mothers' and children's CSA histories predicted children's internalizing and externalizing symptoms. Another paper by Testoni et al. focuses on the relationship between incest and adult female re-victimization treatment within Italian domestic violence centers. The results show that the description of perpetrators was accompanied by psychological and physical abuse. Additionally, the authors report that half of the mothers who did not come to their daughters' aid had also suffered from CSA at a time in their life. These findings support the complex interactions among CSA and other forms of abuse and show the complexities of trauma recovery to be intergenerational and multifaceted. Taken together, these studies are consistent with previous research showing that despite the psychological therapy and agency assistance to victims, the childhood trauma outcomes often remain exhibited in further victimization and violent relationships. It also displays the intergenerational toxic transmission of CSA trauma.

Two papers examine the promise of interventions that include attention to the damage to the brain after CSA. These papers, provided by Hadanny et al. and Lev-Wiesel et al., describe the impact of dual treatment, hyperbaric oxygen treatment (HBOT) and psychotherapy on adult female CSA survivors who also had fibromyalgia (FMS). Efrati's et al. paper shows that following HBOT, there was a significant improvement in FMS and distress symptomatology, demonstrated by a significant increase in brain activity in the prefrontal cortex, orbital frontal cortex, and subgenual area. These results indicate the brain areas in which activity reduction might occur because of CSA. Lev-Wiesel et al.'s, subsequent paper presents qualitative data about the therapeutic process of these female survivors. These data reveal three phases of the dual treatment. The initial phase consisted of remembering the physical and cognitive aspects of the trauma. The second phase included physiological relaxation as well as the emergence of positive memories. The third phase included “bouncing back to life.” These study results seem to support the understanding that physiological and psychological dimensions should be treated concurrently in order to overcome the consequences of CSA trauma.

In our view, the papers presented in this issue illustrate further evidence of the devastating outcomes of CSA and the multidimensional aspects of trauma recovery. They also show the intergenerational complexities of CSA and the relationship of these to both reporting and recovery.

## Author Contributions

As guest editors for this specific issue, RL-W and DS wrote the editorial together. RL-W wrote the first draft of the editorial.

### Conflict of Interest

The authors declare that the research was conducted in the absence of any commercial or financial relationships that could be construed as a potential conflict of interest.
